# Microbiome of Soybean (Glycine max L.) Rhizosphere from Free State, South Africa

**DOI:** 10.1128/mra.00288-22

**Published:** 2022-10-26

**Authors:** Olubukola Oluranti Babalola, Titilope Tinu Ajiboye, Ayansina Segun Ayangbenro

**Affiliations:** a Food Security and Safety Focus Area, Faculty of Natural and Agricultural Sciences, North-West University, Mmabatho, South Africa; SIPBS, University of Strathclyde

## Abstract

Soybean develop a symbiotic relationship with the rhizospheric microbial communities. These organisms are important in maintaining soybean growth and health. Soil samples for this study were collected from Free State, South Africa. We present the microbiome of the soybean rhizosphere and its functional categories at level 1 of the SEED subsystem.

## ANNOUNCEMENT

Plants live in association with rhizosphere organisms, and these organisms perform several functions (1). Rhizosphere organisms fix atmospheric nitrogen, suppress phytopathogens, produce siderophores, and solubilize inorganic phosphates (2). This study examined the taxonomic and functional categories of Link 678 soybean genotype rhizospheric soil. The genotype is widely adaptable in South Africa but is susceptible to drought and root knot nematode. Thus, providing information on the microbiota may facilitate building resilience in the genotype.

Samples were collected from a commercial farm (27.28°S, 26.72°E) in March 2021. Free State is characterized by summer rain, with 500 to 600 mm of rain each year. The summer temperature ranges between 15°C and 32°C, while the winter temperature is between 1°C and 17°C. The field was divided into three regions; from each region, rhizospheric soils attached to the roots (loose soils were discarded) were collected in triplicate (three plants for each sample). Bulk soil samples were collected in triplicate from an uncultivated field 10 m away from the sampling field, at a depth of 5 to 15 cm. Twelve samples were collected, and the samples were kept in an icebox and stored in the laboratory at −20°C. The DNA of the samples was extracted using 0.25 g of each sample with a DNeasy PowerSoil Pro kit (Qiagen, Germany) following the instruction protocol. The Qubit double-stranded DNA (dsDNA) high-sensitivity (HS) assay kit (Life Technologies) was used to determine the DNA concentration. Libraries were prepared using the Illumina DNA Prep (M) Tagmentation library preparation kit according to the manufacturer's protocol. The libraries were made with 20 to 50 ng of DNA. The samples were fragmented, and adaptor sequences were added. These adapters were used in a limited-cycle PCR in which the material was supplemented with unique indices. After library preparation, the final concentrations of the libraries were assessed using the Qubit dsDNA HS assay kit (Life Technologies). The average library size was estimated using the 2100 Bioanalyzer (Agilent Technologies). Subsequently, the libraries were pooled at 0.6 nM equimolar levels and sequenced with paired-end sequencing for 300 cycles on the NovaSeq 6000 system. The sequencing statistics are listed in Table 1.

**TABLE 1:** Statistics for the raw sequences generated in the study

Sample name and sequence SRA accession no.	No. of reads	Total no. of bases	Mean read length (bp)	No. (%) of duplicate reads	GC content (%)
AA					
SRX12237077	24,363,018	3,364,602,389	138.1	328,409 (1.35)	65.55
SRX12237078	20,600,296	2,914,747,038	141.49	259,137 (1.26)	64.96
SRX12237081	17,871,154	2,577,440,961	144.22	212,964 (1.19)	64.86
AB					
SRX12237082	28,092,426	3,803,838,901	135.4	354,811 (1.26)	63.33
SRX12237083	19,030,998	2,745,988,847	144.29	207,368 (1.09)	63.44
SRX12237084	20,235,250	2,844,944,063	140.59	273,087 (1.35)	64.1
CA					
SRX12237085	19,816,690	2,822,298,720	142.42	236,903 (1.2)	63.91
SRX12237086	25,157,886	3,499,754,929	139.11	315,458 (1.25)	64.48
SRX12237087	22,183,110	3,084,046,592	139.03	303,064 (1.37)	64.83
BC					
SRX12237088	18,264,808	2,616,112,978	143.23	228,834 (1.25)	65.53
SRX12237079	17,979,596	2,567,172,719	142.78	222,261 (1.24)	64.99
SRX12237080	20,165,792	2,836,445,764	140.66	243,200 (1.21)	65.46

The sequences were uploaded in the MG-RAST server v4.0.3 (https://www.mg-rast.org) (3). SolexaQA v1.6 was used to perform quality control (QC) on raw data. Dereplication was performed to remove artificial duplicate reads. Duplicate read inferred sequencing error estimation (DRISEE) was performed on the reads to analyze artificial duplicate reads for sequence error reads. The BLAT algorithm (4) was used for annotation with the M5nr database (5), which provides nonredundant integration of multiple databases. All bioinformatic tools used were implemented in MG-RAST with default settings.

The taxonomic assignments based on the M5nr database showed that *Proteobacteria*, *Actinobacteria*, *Firmicutes*, *Acidobacteria*, and *Bacteroidetes* were the dominant bacterial phyla in all samples. Ascomycota and Basidiomycota were the dominant fungal phyla, while *Euryarchaeota*, *Crenarchaeota*, and *Thaumarchaeota* were the dominant archaeal phyla in all samples.

Twenty-eight functional categories were assigned using the SEED subsystem (6). The categories include carbohydrates, amino acids and derivatives, protein metabolism, membrane transport, cell wall and capsule, and nucleosides and nucleotides, among others.

### Data availability.

The BioProject accession number is PRJNA763981, and the Sequence Read Archive (SRA) accession numbers are as follows: for the bulk soil samples (sample BC): SRX12237079, SRX12237080, and SRX12237088; for the rhizosphere soil samples: sample AA, SRX12237077, SRX12237078, and SRX12237081; sample AB, SRX12237082, SRX12237083, and SRX12237084; sample CA, SRX12237085, SRX12237086, and SRX12237087.
